# Glutathione Metabolism in Renal Cell Carcinoma Progression and Implications for Therapies

**DOI:** 10.3390/ijms20153672

**Published:** 2019-07-26

**Authors:** Yi Xiao, David Meierhofer

**Affiliations:** 1 Max Planck Institute for Molecular Genetics, Ihnestraße 63-73, 14195 Berlin, Germany; 2 Freie Universität Berlin, Fachbereich Biologie, Chemie, Pharmazie, Takustraße 3, 14195 Berlin, Germany

**Keywords:** Renal cell carcinoma (RCC), reactive oxygen species (ROS), glutathione (GSH) metabolism, cancer therapy, clear cell RCC, papillary RCC, chromophobe RCC

## Abstract

A significantly increased level of the reactive oxygen species (ROS) scavenger glutathione (GSH) has been identified as a hallmark of renal cell carcinoma (RCC). The proposed mechanism for increased GSH levels is to counteract damaging ROS to sustain the viability and growth of the malignancy. Here, we review the current knowledge about the three main RCC subtypes, namely clear cell RCC (ccRCC), papillary RCC (pRCC), and chromophobe RCC (chRCC), at the genetic, transcript, protein, and metabolite level and highlight their mutual influence on GSH metabolism. A further discussion addresses the question of how the manipulation of GSH levels can be exploited as a potential treatment strategy for RCC.

## 1. Introduction

**Figure 1:**
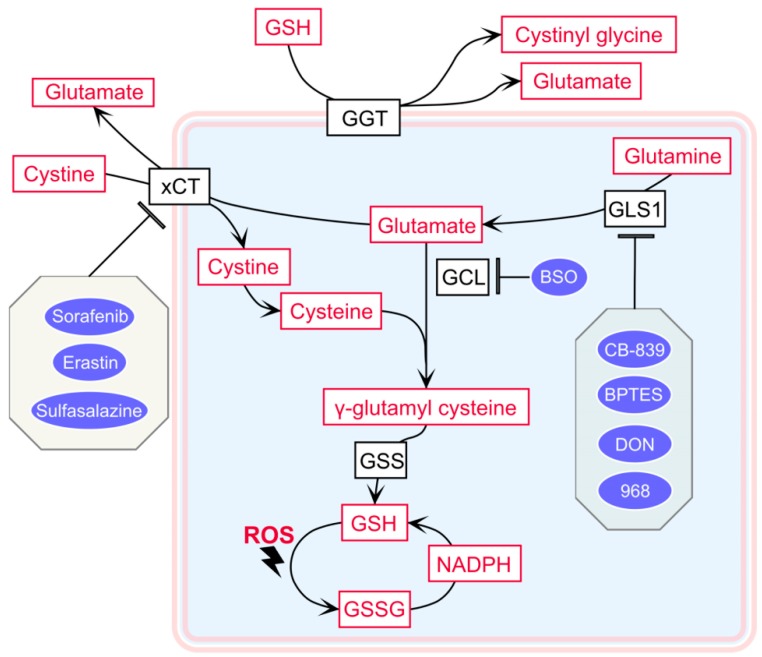
Schematic overview of glutathione (GSH) metabolism and the targeting sites of inhibitors. Color codes are defined as follows: black = enzymes or transporters; red = metabolites; blue = inhibitors. GGT: γ-glutamyl transferase; xCT: solute carrier family 7 member 11, a cystine-glutamate antiporter; GLS1: glutaminase 1; GCL: glutamate cysteine ligase; GSS: glutathione synthetase. GSSG: glutathione oxidized form; ROS: reactive oxygen species.

Increased reactive oxygen species (ROS) levels, including the superoxide anion, hydrogen peroxide, and hydroxyl radical, have been reported in many different cancer types. ROS can be either generated by genetic alterations and endogenous oxygen metabolism or by exogenous sources, such as UV light and radiation. ROS were long thought to be only damaging byproducts of the cellular metabolism that can negatively affect DNA, lipids, and proteins [1]. However, more recent studies have highlighted the important role of ROS in cell signaling, homeostasis, metabolism, and apoptosis [1]. One common characteristic of cancer is the ability to balance the increased level of oxidative stress with a high level of antioxidants. Glutathione (GSH), a tripeptide thiol antioxidant composed of the amino acids glutamic acid, cysteine, and glycine [2], is the main ROS scavenger in cells. GSH is highly reactive and exists in both a reduced (GSH) and oxidized disulfide (GSSG) form [3]. The predominant form is in the reduced state, which is the most abundant low molecular weight thiol in the cell, ranging from 0.5 to 10 mM in most cell types, whereas extracellular GSH exists in concentrations lower by magnitudes [4]. The de novo biosynthesis of GSH involves two ATP-dependent enzymatic reactions: The first step is catalyzed by glutamate cysteine ligase (GCL), which ligates the amino group of cysteine to the γ-carboxylate of glutamic acid to form the dipeptide γ-glutamyl cysteine. The second reaction involves GSH synthetase (GSS), which catalyzes a combination of the cysteinyl carboxylate of the dipeptide and the amino group of glycine to synthesize GSH [5] (outlined in Figure 1).

Besides the classical role of GSH acting as an ROS scavenger by being prey for radicals, GSH has several additional functions, including but not limited to providing a cysteine reservoir [6], being involved in the maturation of iron–sulfur proteins [7], detoxifying xenobiotics [8], regulating protein bioactivity by S-glutathionylation [9,10], and regulating redox signaling [11]. In cancer, GSH plays the role of a double-edged sword in its initiation and progression. Moderate ROS levels are widely recognized to trigger cancer initiation and progression by inducing mutations and promoting genome instability, eventually activating oncogenic signaling pathways that promote cell survival, proliferation, and stress resistance [12]. On the contrary, massive ROS accumulations can also limit cancer growth by causing severe oxidative damage of biomolecules, which finally can lead to cell death [13]. As a consequence, cancer cells are required to deliberately balance the levels of ROS and antioxidants (mainly GSH) to maintain redox homeostasis, which sustains viability and growth. For many years, one of the most obvious therapeutic strategies to overcome new balanced redox homeostasis in renal cell carcinoma (RCC) was to fight elevated ROS levels with the supplementation of antioxidants such as vitamins to actively force the tumor into apoptosis. Many clinical trials were initiated, and the outcomes showed mixed results, including worse survival rates upon supplementation with ROS inhibitors [14].

In this review, we will focus on the role of the ROS scavenger GSH in RCC and discuss possible strategies that can potentially exploit the manipulation of GSH levels for therapeutic strategies in RCC.

## 2. Renal Cell Carcinoma: An Overview

RCC represents approximately 4% of adult malignancies [15] and was ranked as the sixth deadliest cancer worldwide in 2018 [16]. The American Cancer Society estimated that about 73,820 new RCC cases would be diagnosed by the end of 2019 and more than 14,770 deaths would be caused by RCC this year in the USA alone [15]. RCC can be classified according to distinct morphologic and molecular genetic features and is composed of different subtypes, such as clear cell RCC (ccRCC), papillary RCC (pRCC), and chromophobe RCC (chRCC, Table 1). Many studies have been performed recently to characterize RCC to better understand its classification and subclassification and to elucidate pathway remodeling in these cancers [17,18,19]. A new classification concept based on molecular clustering of chromosoms, DNA, RNA, miRNA, and protein data was proposed [18,20], where the organ of origin does not fully determine the tumor type as the only factor [21]. Instead, the cancer classification should be based on the similarity of molecular features across different tissue types, which was considered to be more relevant for targeting the same mutations and oncogenic signaling pathways [21].

Here, we review the genetic foundation of the main RCC types and shed light on the sparse data of transcriptome, proteome, and metabolome profiles performed in these malignancies.

### 2.1. Clear Cell Renal Cell Carcinoma

Clear cell RCC is the most prevalent subtype and accounts for about 75% of all RCCs (Table 1) [22]. It is an aggressive cancer that originates from the proximal convoluted tubule, with a recurrence rate of up to 40% after the initial treatment of a localized tumor [23]. In its metastatic form, it is associated with a high mortality rate [24]. Clear cell RCC cells have, in general, a clear cytoplasm, (which helped coin the name “clear cell”) that is circled by an easily distinguishable cell membrane and uniform round nuclei [25]. About 90% of all ccRCCs carry mutations in the von Hippel-Lindau (*VHL*) tumor suppressor gene [17,26], which was originally identified in a hereditary disease called VHL syndrome [27]. The VHL protein is a target recruitment subunit in an E3 ubiquitin ligase complex and recruits the hydroxylated hypoxia-inducible factor (HIF) under normoxic conditions for subsequent proteasomal degradation. Thereby, VHL can repress the transcription of more than 100 target genes through interaction with HIF1α and HIF1AN, which plays a vital role in forming the phenotype of ccRCC [28]. HIF1α is a master transcription factor that contributes substantially to the regulation of gene expression that is dependent on oxygen levels. Under normoxic conditions, VHL interacts with HIF1α and hydroxylates the proline residues in the oxygen-dependent degradation (ODD) domains of HIF1α by recruiting members of the Egl-nine homolog (EGLN) family [28,29,30]. With hypoxia or loss of function of VHL, these proline residues cannot be hydroxylated, which stabilizes HIF1α. HIF1α subsequently forms a HIF1α–HIF1β heterodimer, and this dimer translocates into the nucleus to enhance the transcription of HIF target genes, which are associated with crucial oncogenic pathways, including glucose uptake, glycolysis (e.g., glucose transporter type 1, *GLUT1*), cell proliferation (e.g., epidermal growth factor receptor, *EGFR*), and angiogenesis (vascular endothelial growth factor, *VEGF*) [30,31,32,33].

Furthermore, the gluconeogenic enzyme fructose 1,6-bisphosphatase 1 (FBP1) has been found to be decreased in over 600 ccRCCs and has been associated with poor disease prognosis. FBP1 has two distinct functions, antagonizing the glycolytic flux and inhibiting the nuclear function of HIFα [34], which can explain its ubiquitous loss in ccRCC [34]. Besides FBP1, the whole gluconeogenesis pathway has been shown to be severely diminished in ccRCC at the transcriptome [34] and proteome level [35]. This stimulates the metabolic switch by increasing glycolytic target genes [34], which is reflected by the metabolomic analysis of ccRCC, where metabolites in the glycolysis pathway show over two-fold increases in abundance compared to the normal kidney [36]. Furthermore, GSH metabolism-related metabolites, including cysteine, γ-glutamyl cysteine, and GSH, have all been shown to increase in late-stage ccRCC and are associated with worse survival outcomes in ccRCC patients [36].

### 2.2. Papillary Renal Cell Carcinoma

Papillary RCC represents about 15% of all RCCs (Table 1) and also derives from the proximal convoluted tubule, similarly to ccRCC [22]. It is a less aggressive subtype compared to ccRCC and has a high five-year survival rate of 80% to 85% [37]. The term “papillary” describes the papilla-like protuberances in most of the tumors. It can be further subdivided into type I and type II tumors based on morphological features. Type I pRCC is more common and shows small fibrovascular papillae that are covered by a single layer of small cuboidal cells with scant pale cytoplasm and usually grows slowly [38]. In contrast, type II pRCC consists of papillae, is lined by large columnar pseudostratified cells with an eosinophilic cytoplasm, and is often more aggressive [38,39]. Type I and type II pRCC have also been shown to be clinically and biologically distinct, as alterations in the MET pathway were associated with type I [18,40]. The proto-oncogene c-Met (MET) protein, a transmembrane receptor tyrosine kinase, can bind to its ligand hepatocyte growth factor (HGF) and activate several downstream intracellular pathways, including focal adhesion kinase (FAK), RAS/RAF/MEK/ERK, and PI3K/AKT [41]. The frequently activating mutations and amplification of *MET* in type I pRCC enable the activation of MET/HGF signaling and its above-mentioned downstream pathways to promote cancer cell proliferation, angiogenesis, and malignant transformation [41].

Frequent mutations in type II pRCC include *CDKN2A* silencing, *SETD2* mutations, and *TFE3* fusions. Type II tumors are characterized by increased expression of the nuclear factor erythroid 2-related factor 2 (*NRF2*)–antioxidant response element (ARE) pathway [18]. The NRF2–ARE pathway is a major regulator of cellular redox balance, and its activation under oxidative stress favors cell survival. Furthermore, fumarate hydratase (*FH*) mutations are also frequently found in type II pRCC [42,43]. The *FH* gene encodes a TCA cycle enzyme that catalyzes the hydration of fumarate to malate, and its deficiency causes fumarate and succinate accumulation [44,45]. Accumulated fumarate and succinate are believed to be able to suppress the hydroxylation of the proline residues in the ODD domain of HIFα, and thus *FH* mutations in type II pRCC also cause the stabilization of HIFα, similarly to ccRCC [44,45]. Some genes (such as *CDKN2A/B* and *TERT*) where mutations can be found in both types [40] play a pivotal role as tumor suppressors by regulating the cell cycle. Mutations of the above-mentioned genes and activation of the oncopathways are the main driver mutations in the progression of pRCC.

How do these genetic alterations in pRCC translate to the protein and metabolite level? Proteome profiles of pRCC versus matching healthy tissues have indicated a tremendous reprogramming of main metabolic pathways. Oxidative phosphorylation, the TCA cycle, branched-chain amino acids, cytochrome P450 drug metabolism, peroxisomes, fatty acid metabolism, and several amino acid metabolism pathways were significantly decreased in pRCC, whereas the spliceosome, the ribosome, and the cell cycle were significantly increased [46]. A striking anticorrelation between the proteome and the transcriptome data [18] was identified for oxidative phosphorylation. Transcripts of the respiratory chain were significantly increased in pRCC, whereas the entire pathway was significantly decreased on the proteome level. Most likely, the lower protein abundance of the respiratory chain was a consequence of the reduced mitochondrial DNA (mtDNA) content in pRCC, as a similar association was observed in ρ^0^ cells [47]. ρ^0^ cells entirely lacking mtDNA, which encodes for 13 core respiratory chain subunits and consequently miss all of the respiratory chain complexes [47]. Furthermore, the discrepancy between the transcripts and proteins of the respiratory chain in RCC can also originate from the regulation of post-translational modifications. The metabolome indicated a tremendous increase of reduced and oxidized GSH levels in pRCC tissues [46], as well as significantly increased rates of GSH de novo synthesis based on glutamine consumption in the pRCC-derived cell lines Caki-2 and ACHN [46]. All of these alterations can in principle serve as potential therapeutic targets. Specifically, a dysregulated respiratory chain can cause electron leakage [48,49]. This, in turn, leads frequently to an increase in ROS stress [50,51], which is subsequently compensated for by increased GSH levels in RCC and might serve as a main therapeutic site to eradicate RCC, as discussed later on.

### 2.3. Chromophobe Renal Cell Carcinoma

Chromophobe RCC accounts for approximately 5% of all RCCs (Table 1) [22], is thought to originate from the cortical collecting duct, and was first reported in 1985 [52]. Different morphological and ultrastructural features of the cytoplasm lead to the identification of the classical chromophobe and the eosinophilic variant. The cells of the classical type usually have abundant clear cytoplasm and a perinuclear halo caused by cytoplasmic organelles being pushed away from the center to form a rim along the cell membrane [53]. The eosinophilic type has, in general, smaller cells with an inconstant level of cytoplasmic organelles in the periphery. Both cell types frequently coexist in chRCC tumors, usually with one cell type predominating [53]. One of the most characteristic genetic features of chRCC is the monosomy of chromosomes 1, 2, 6, 10, 13, 17, and often 21 [54,55,56,57]. The most commonly mutated genes in chRCC are *TP53* (32%), *PTEN* (20%), and gene fusions involving the *TERT* promoter [19,54]. Mutations in these tumor suppressors combined with the deletion of one of their chromosomes leads to a complete loss of function. Further mutations with a lower frequency were observed in *MTOR*, *NRAS*, *TSC1*, and *TSC2*, indicating that the genomic targeting of the mTOR pathway occurred in 23% of all chRCC [19]. Hence, the anticancer functions of TP53 in apoptosis, genomic stability, and the inhibition of angiogenesis and the role of PTEN in the intracellular signaling pathway PI3K/AKT/mTOR are both disrupted and can thus be regarded as major driving events in chRCC tumorigenesis.

Proteome profiling has identified metabolic reprogramming in chRCC, including stalled gluconeogenesis, downregulated oxidative phosphorylation, and fatty acid and amino acid metabolism [57]. A similar anticorrelation between transcripts and proteins (as in pRCC) was also identified in chRCC. As chRCC has a significantly lower microvessel density and a lower glucose uptake rate compared to ccRCC and pRCC [58,59], it seems that chRCC cells prefer a different way to acquire nutrients to compensate for the nutrient-poor microenvironment. Chromophobe RCC cells can activate the endocytosis and downstream lysosomal pathways to gain extracellular macromolecules as a nutrition source for cell survival and proliferation, which is indicated by the abundance increase of proteins involved in these pathways and their enzymatic activities [57]. Metabolome profiling in chRCC [57,60] and the closely related but hardly distinguishable benign renal oncocytomas [61,62] has also elucidated a striking increase of GSH and GSSG levels in kidney tumors, thus a hallmark in all RCCs.

In the next chapters, we will investigate the role of GSH metabolism in RCC progression and how this adaption to increased ROS levels can be exploited therapeutically.

## 3. Rewired Glutathione Metabolism in RCC Is a Key Metabolic Alteration Involved in Tumor Progression

### 3.1. γ-Glutamyl Cycle and ccRCC Progression

The γ-glutamyl cycle was originally proposed by Meister in 1970 and involves the de novo biosynthesis and degradation of GSH [63]. Its main functions rely on the two enzymes GCL and GSS for biosynthesis and γ-glutamyl transferases (GGTs) for degradation (Figure 1).

GCL is involved in the first step of de novo GSH synthesis, which catalyzes the reaction of γ-glutamyl cysteine production. Other than substrate availability, GCL is the rate-limiting enzyme of GSH biosynthesis. It is composed of two different subunits: GCLC, the 73-kD catalytic subunit, which contains the active site for catalyzing the reaction; and GCLM, the 31-kD modulatory subunit, which interacts with GCLC to increase catalytic efficiency [64]. Under physiological conditions, the activity of the GCLC/GCLM heterodimer can be regulated by a negative feedback loop by its product GSH [5]. GCLC and GCLM were reported to have increased protein abundances, matching the significantly increased enzymatic activity in the tumor tissues of ccRCC patients [34,65,66]. Validation by siRNA-mediated silencing of GCLC led to a strong cell number reduction of ccRCC cell lines, which was accompanied by significantly decreased GSH levels [66]. The direct inhibition of GSH synthesis caused ferroptosis, a nonapoptotic form of cell death, in ccRCC cells [66]. These data support the substantial role of GSH metabolism in ccRCC progression.

GGTs are membrane-bound, N-terminal nucleophile hydrolases that catalyze the breakdown of extracellular GSH and transfer the γ-glutamyl group from GSH to produce the constituents glutamate and cysteine, which can be further used for intracellular GSH synthesis (Figure 1) [67]. Increased serum GGT was reported to be a sensitive marker for metastatic ccRCC [68], as GGT levels positively correlated with advanced stages, higher grades, and the presence of tumor necrosis, and it was further associated with worse survival rates in ccRCC patients [69].

### 3.2. Precursor Amino Acid Availability for GSH de novo Synthesis

**Figure 2:**
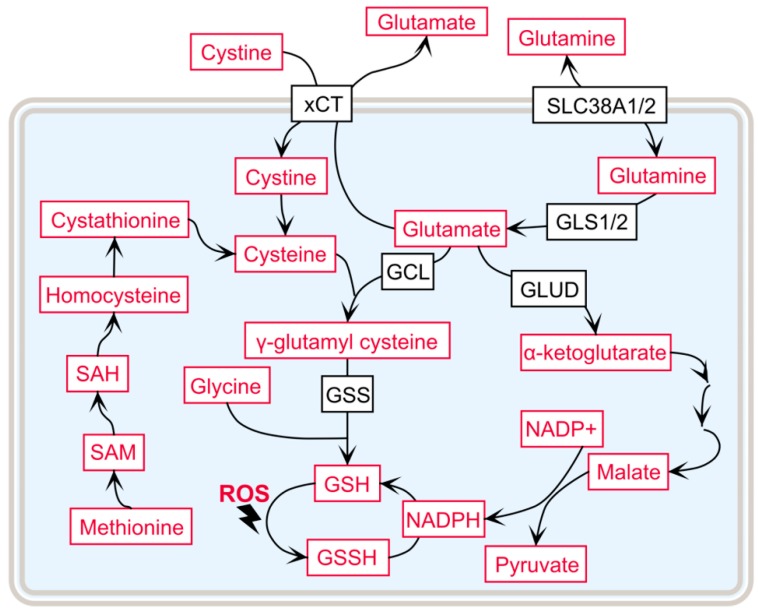
The availability of the precursor amino acids that influence GSH synthesis. Color codes are defined as follows: black = enzymes or transporters; red = metabolites. SLC38A1/2: solute carrier family 38 member 1 and 2, glutamine transporters; GLUD: glutamate dehydrogenase; SAM: S-adenosyl methionine; SAH: S-adenosyl homocysteine.

Apart from the γ-glutamyl cycle, GSH de novo synthesis also relies on the availability of its three composing amino acids—glutamate, cysteine, and glycine—and the activity of their corresponding transporters, as summarized in Figure 2.

One of the metabolic hallmarks of ccRCC is the addiction to glutamine. The malignancies, therefore, require exogenous glutamine for growth and feature reprogrammed glutamine metabolism [70,71]. The availability of glutamine can directly or indirectly influence GSH de novo synthesis in three different ways. First, glutamine can be converted to glutamate by two isozymes, glutaminase 1 and 2 (GLS1 and GLS2). GLS1 is increased in many cancer types and is the main isoform within the kidney [72]. Second, glutamine-related transporter transcripts are consistently increased in ccRCC tumors, e.g., the glutamine importers *SLC38A1* and *SLC38A2* [73], to sustain glutaminolysis in ccRCC. Interestingly, SLC38A1 expression is also regulated by MYC in K562 and HeLa cells: Considering that MYC has been shown to be upregulated and that MYC pathway activation cooperates with VHL loss to induce ccRCC [73,74,75], SLC38A1 probably plays an important role in the progression of ccRCC. Third, glutamine can contribute to the de novo synthesis of GSH through the generation of NADPH via glutamate dehydrogenase (GLUD) or malate regulation [76]. Overall, glutamine is of vital importance to GSH de novo synthesis to modulate the oxidative stress level in ccRCC.

Cysteine, although a nonessential amino acid, plays an important role in protein synthesis by forming intraprotein disulfide bonds to stabilize proteins. There are multiple cellular pathways and transporters, which contribute to the availability of cysteine in cells. Aside from the γ-glutamyl cycle, the trans-sulfuration pathway serves as an important source for cysteine recruitment, which has been reported to be dysregulated in ccRCC [34,36]. Thereby, methionine is converted to S-adenosyl methionine (SAM), is hydrolyzed to homocysteine, and can then enter the trans-sulfuration pathway, where it gets converted into cystathionine, which forms cysteine in subsequent reactions [77]. Metabolome profiling of ccRCC tumors has identified elevated SAM, S-adenosyl homocysteine (SAH), and homocysteine in ccRCC compared to healthy kidney tissues [34,36], indicating a high demand for cysteine, which is synthesized through the trans-sulfuration pathway. Cystine, the oxidized dimer of cysteine, can also be absorbed from the tumor microenvironment by xCT (*SLC7A11*), which is a heterodimeric cystine–glutamate antiporter [78], where overexpression is associated with overall poor survival in ccRCC [26].

### 3.3. Increased Flux of the Pentose Phosphate Pathway in ccRCC to Support GSH Synthesis

The pentose phosphate pathway (PPP) is, in part, a metabolic pathway parallel to glycolysis. It generates NADPH and pentoses, including ribose 5-phosphate (R5P). Its primary role is considered to be anabolic rather than catabolic, as it provides R5P, a precursor for the synthesis of nucleotides. NADPH is an important cofactor for the enzyme GSH reductase (GR) to catalyze the reduction of GSSG to GSH and hence links PPP directly to GSH synthesis. Therefore, high GSH/GSSG ratios in ccRCC can also be explained by increased flux through PPP, which provides the necessary molecules of NADPH for GSH conversion [26,34,79]. Indeed, glucose-6-phosphate dehydrogenase (G6PD), which determines the production of NADPH and R5P within PPP, was found to be increased in ccRCC, and its elevation was associated with higher levels of NADPH and PPP-derived metabolites [79]. Furthermore, the inhibition of G6PD in chRCC cells decreased the NADPH level and increased ROS production to significantly impair cancer cell survival, suggesting that PPP plays a fundamental role in the regulation of redox homeostasis and progression in ccRCC [79]. Recently, fructose 1,6-bisphosphate (FBP), a glycolytic intermediate, was found to be accumulated in ccRCC, leading to the suppression of NADPH oxidase 4 (NOX4), which caused an increase in NADPH and a decrease in ROS, independent of PPP [80]. These changes were caused by the downregulation of aldolase B (ALDOB), which portended significantly worse survival in ccRCC patients [80].

### 3.4. Fumarate Hydratase Mutations and GSH in Type II pRCC

Fumarate hydratase (*FH*) is frequently mutated in type II pRCC, causing FH deficiency and affecting the normal flux of the TCA cycle [42,43]. The FH deficiency of this tumor leads to metabolic reprogramming, including impaired oxidative phosphorylation and aerobic glycolysis, known as the “Warburg effect”, which is probably caused by the stabilization of HIF through fumarate accumulation [44,45]. A further study showed that the accumulation of fumarate was fueled by glutamine rather than glucose in type II pRCC cells [81]. Apart from causing HIF stabilization, fumarate accumulation has been reported to activate NRF2 and its downstream ARE pathway [82,83]. Moreover, somatic mutations of *NRF2* and its regulator Kelch-like ECH-associated protein 1 (*KEAP1*) have been reported to be highly correlated with poor prognosis in type II pRCC and pancreatic cancer [82,84,85]. NRF2 plays an important role in cellular redox balance as a transcription factor that regulates the expression of various genes to combat the harmful effects of extrinsic and intrinsic damage, such as xenobiotics and oxidative stress. NRF2 is primarily regulated by KEAP1, a substrate adapter protein of Cullin 3 (CUL3), which contains an E3 ubiquitin ligase activity. Under normal conditions, NRF2 builds a complex with KEAP1 via its Kelch domain for ubiquitination, and NRF2 is then targeted for subsequent proteasomal degradation [84,86,87]. However, in response to a diverse array of stimuli, such as oxidative stress, the cysteine residues within KEAP1, Cys151, Cys273, and Cys288 can be modified, which results in a conformational change along with the dissociation of NRF2 to avoid KEAP1-mediated degradation [84,87]. Stabilized NRF2 can then translocate to the nucleus and bind to ARE to activate the downstream effector genes of at least two pathways involved in cytoprotection [84]. First, NRF2 can activate genes involved in regulating GSH synthesis and metabolism by activating GCL [85], and second, NRF2 promotes the expression of genes coding for antioxidant proteins, such as GSH peroxidases (GPXes) and GSH S-transferases (GSTs). GPX is an enzyme family with peroxidase activity that protects cells from oxidative damage, and GSTs are comprised of a family of isozymes that catalyze the conjugation of GSH to xenobiotic substrates for detoxification [78,84]. Additionally, fumarate can directly bind to GPX1 through interaction with the Thr143 and Asp144 residues, and fumarate accumulation is thus able to activate GPX1 and decrease the ROS level in cells [88].

Apart from mutations in *FH*, *NRF2*, *CUL3*, or *KEAP1* in type II pRCC, several other mechanisms can also lead to increased NRF2 activity in other cancers, including epigenetic silencing, modifications of cysteine residues, metabolic alterations, and oncogene-dependent signaling [85].

### 3.5. Glutathione Salvage Pathway in chRCC

One member of the membrane transpeptidase family GGT is γ-glutamyl transferase 1 (GGT1), which can remove and transfer the γ-glutamyl moiety from extracellular GSH, GSSG, or even GSH conjugates to an amino acid acceptor, known as the GSH salvage pathway. This degradation of extracellular GSH species fuels the cytoplasm of cells to maintain intracellular GSH levels [67]. Recent metabolomic profiling studies have identified significantly increased amounts of GSH, GSSG, and its precursor γ-glutamyl cysteine in chRCC compared to normal kidney tissue [57,60]. Unlike in ccRCC, significantly lower expression of GGT1 has been reported in chRCC [57,60]. The specific loss of GGT1 in chRCC leads to an increased sensitivity to oxidative stress, mitochondrial damage, and reprogramming of glutamine and glucose metabolism [60]. Interestingly, renal oncocytomas, which are considered to be the benign counterpart of chRCC, were found to have a similar increase of GSH moieties and decreased levels of GGT1 relative to normal kidney tissue [61].

## 4. Therapeutic Strategies to Exploit Increased GSH Levels in RCC

Nonmetastatic primary RCC can be removed by partial or complete nephrectomy. Metastases that occur in about one-third of all RCC patients must be treated with various therapeutic agents [89]. One severe problem is that the malignancies gain a fast treatment resistance through the activation of alternative metabolic pathways, or parts of the cancer cells that are not responsive outgrow the responsive tumor cells. For example, angiopoietin 2, MET, or Interleukin (IL) can serve as alternative angiogenesis factors, or the AKT/PI3K/mTOR pathway can stimulate proliferation upon its activation [90,91]. Depletion of GSH alone has been shown to be insufficient to induce cell death in most cancer cell lines. The imposed selective pressure during cancer initiation and progression led to a robust adaption mechanism to tolerate these stress conditions [92]. To overcome these limitations, combinatory therapies targeting two independent mechanisms are promising. The following subchapters highlight current strategies that manipulate GSH metabolism and intend to eradicate RCC.

### 4.1. The Cystine–Glutamate Shuttle Inhibitor

xCT is a cystine–glutamate antiporter that is essential to the uptake of cystine (Figure 1). After the conversion of cystine into cysteine, it serves as a building block for the synthesis of intracellular GSH, as discussed before. xCT is upregulated in a variety of cancers, where the antiporter-assisted production of GSH reduces oxidative stress levels to protect cancer from apoptosis [93]. Pharmacological inhibition of xCT decreases cystine uptake and induces ferroptosis in cancer cells [94], which makes xCT inhibitors potential treatment agents for cancer.

Sorafenib, an FDA-approved kinase inhibitor drug used for almost 15 years for the treatment of RCC, has multiple kinase inhibition activities, including cell surface tyrosine kinases (e.g., vascular endothelial growth factor receptor, VEGFR; platelet-derived growth factor receptor, PDGFR; tyrosine-protein kinase kit, KIT; Fms-like tyrosine kinase 3, FLT3; RET proto-oncogene, RET) and downstream intracellular serine/threonine kinases (e.g., both wild-type and mutant BRAF and CRAF) [95]. As these kinases play important roles in cancer cell proliferation, angiogenesis, and apoptosis, sorafenib has been shown to inhibit the proliferation of cancer cells and induce apoptosis in vitro, as well as reduce angiogenesis and inhibit tumor growth in vivo [95]. Recently, sorafenib, but not other kinase inhibitors of the same class, has been reported by several studies to have novel inhibition activity versus xCT, leading to decreased cysteine uptake, GSH depletion, and ROS accumulation, finally causing endoplasmic reticulum stress and ferroptosis [94,96,97].

Besides sorafenib, there are two other xCT inhibitors worth discussing in more detail, erastin and sulfasalazine. Erastin is a small molecule that inhibits xCT activity through the mitochondrial voltage-dependent anion channel 2 and 3 (VDAC2 and VDAC3), causing abolition of the antioxidant defenses of the cell, and it furthermore has selectively lethal activity toward oncogenic *RAS* mutant cell lines [98,99]. A cystine addiction of VHL-deficient RCC cells was identified, and the deprivation thereof or treatment with erastin or sulfasalazine in RCC cells induced cell death [100]. Chromophobe RCC, but not pRCC, was found to have significantly increased abundances of the VDAC1, VDAC2, and VDAC3 proteins [46,57] and should have a good response to treatment with erastin. Sulfasalazine has been in use for over 50 years for the treatment of inflammatory conditions such as arthritis. It is a well-characterized specific inhibitor of xCT and shows anticancer effects on multiple types of cancers, including RCC [94,98,100]. The safety and side effects of sulfasalazine are well investigated and understood, and this old drug has the potential to be a novel, effective, and economical treatment option for RCC patients.

### 4.2. Glutaminase 1 Inhibitor

Glutaminase 1 (GLS1), a key mitochondrial enzyme that controls glutamine metabolism and contributes to de novo GSH synthesis (Figure 1), is very important for tumor proliferation and survival. The glutaminase inhibition of glutamine-addicted cancer cells leads to the disruption of metabolic pathways, such as macromolecule synthesis, ATP production, and the intracellular redox balance [101]. Thus, targeting glutaminase to disrupt vital metabolic pathways of tumors is considered to be a novel strategy to treat cancer.

CB-839, a potent, selective, and orally bioavailable GLS1 inhibitor, has been reported to exhibit significant antiproliferative activity in multiple cancer cell lines and has shown an antitumor effect in tumor xenografts and cancer patients [102]. CB-839 is currently being investigated in multiple phase 1 and 2 clinical trials for patients with locally advanced, metastatic, and/or refractory solid tumors, including ccRCC [102]. Emberley et al. [102] reported a cytotoxic effect of CB-839 in 18 out of 23 tested RCC cell lines and 0 out of 6 non-RCC cell lines. MacKinnon et al. [103] found that the abundance of pyruvate carboxylase (PC), which catalyzes the conversion of pyruvate to oxaloacetate to fuel the TCA cycle, strongly correlated with resistance, and knockdown of PC reduced TCA cycle activity and sensitized cells to CB-839 treatment, suggesting that PC expression may be a biomarker of resistance to CB-839. Chromophobe RCC and pRCC have been reported recently to have low PC expression [46,57]: They may lack this mechanism and therefore would probably be sensitive to CB-839. In addition, decreases in mTOR signaling were also observed in RCC cell lines that were sensitive to CB-839, indicating that CB-839-induced glutamate deprivation has a direct influence on the mTOR pathway [102]. These observations suggest that receptor tyrosine kinase (RTK) signaling or mTOR inhibitors would have synergistic effects with CB-839 to increase cytotoxicity in RCC cell lines. The combined CB-839 and cabozantinib (RTK inhibitor) therapy, which reached phase 2 clinical evaluation (CANTATA: NCT03428217), showed pronounced reductions in TCA cycle activity and in signaling via AKT and ERK compared to single-agent treatments. When applying CB-839 in combination with everolimus (mTOR inhibitor) to RCC cell lines in vitro and to a Caki-1 RCC xenograft model in vivo, synergistic antitumor activity and inhibition of both glucose and glutamine utilization were observed [104]. Furthermore, the combined therapy of CB-839 with everolimus in a phase 1 clinical trial showed a 100% disease control rate (DCR) in ccRCC and 67% in pRCC [104], and it is currently in a phase 2 investigation in patients with advanced ccRCC (ENTRATA: NCT03163667). Moreover, a phase 1/2 study of CB-839 in combination with nivolumab (anti-PD-1 antibody) is currently ongoing (NCT02771626) and has shown a 74% DCR in ccRCC patients. Furthermore, CB-839 was found to have a synergistic effect in selectively suppressing the growth of ccRCC cells in vitro and in vivo when combined with poly(ADP-ribose) polymerase (PARP) inhibitors [105]. Currently, a phase 1b/2 clinical trial of CB-839 in combination with talazoparib (PARP inhibitor) is under investigation (NCT03875313).

At present, CB-839 is the only small-molecule GLS1 inhibitor being studied in a clinical setting, but there are other GLS1 inhibitors in preclinical investigations, including bis-2-(5-phenylacetamido-1,2,4-thiadiazol-2-yl)ethyl sulfide (BPTES), 6-diazo-5-oxo-l-norleucine (DON), and 5-[3-bromo-4-(dimethylamino)phenyl]-2,3,5,6-tetrahydro-2,2-dimethyl-benzo[a]phenanth-ridin-4(1H)-one (968). Two of these GLS1 inhibitors have not been further applied and investigated in clinical studies due to the low solubility and potency of BPTES and the high toxicity and poor binding selectivity of DON [106]. However, 968 is known to be a noncompetitive inhibitor of GLS1 and is currently still in the preclinical stage [106].

### 4.3. The Glutamate–Cysteine Ligase Inhibitor Buthionine Sulfoximine 

GCL, the enzyme catalyzing the first reaction in GSH de novo synthesis (Figure 1), plays an important function in maintaining intracellular GSH levels to combat oxidative stress in RCC [65,66]. Thus, targeting GCL for the treatment of RCC remains a potentially effective strategy to benefit patients.

Buthionine sulfoximine (BSO) is a specific and competitive inhibitor of GCL [107]. Developed in 1979 by Griffith and Meister [108], BSO is able to decrease the intracellular GSH level and sensitize different types of cancers both in vitro and in vivo to various chemotherapies and other cytotoxic therapies, e.g., irradiation and hyperthermia [107,109]. BSO has been reported to enhance the activity of melphalan, doxorubicin, daunorubicin, and other cytotoxic agents in myeloma, breast cancer, and lung cancer [107,109]. Two clinical trials of BSO in combination with melphalan were conducted for the treatment of neuroblastoma. In a pilot study and a phase I clinical trial, BSO in combination with melphalan was well tolerated and had therapeutic activity toward recurrent and refractory high-risk neuroblastoma (NCT00002730, NCT00005835) [110,111]. Sorafenib, as discussed earlier, has xCT and multiple kinase inhibition activity. However, it has been reported that some RCC patients were initially resistant or acquired resistance to sorafenib within a median of 5–9 months [112]. Mechanistic studies have shown that the resistance to sorafenib in RCC was mediated by enhanced expression of *HIF* and numerous *HIF*-regulated genes, such as *VEGF* [112,113]. As the redox state could regulate *HIF* expression and downstream substrates to cause drug resistance [29,114], BSO, which can regulate the redox environment through GSH, was shown to be able to decrease the expression of *HIF* [114,115,116]. These studies indicate that combination therapies with BSO and sorafenib might overcome the drug resistance of sorafenib in resistant RCC patients.

### 4.4. Inhibition of Deubiquitinating Enzymes Initiates Proteotoxicity

A recent report outlined a combinatory treatment strategy applying the before-mentioned GCL inhibitor BSO together with deubiquitinating enzyme (DUB) inhibitors [92]. Individual DUB inhibitors, such as MI-2, PR-619, and EERI, were not effective in inducing cell death alone but led to an induction of proteotoxic stress and cell death in combination with BSO in many different cancer cell lines. Though the exact molecular role of how DUBs can protect cells from oxidative stress is still elusive, a dependency on DUB activity to maintain protein homeostasis by eliminating the accumulation of damaged and potentially cytotoxic polyubiquitinated proteins and cell viability have been proposed [92]. This hypothesis was supported by a study profiling the ubiquitination status between ρ^0^ cells, which entirely lack mitochondrial DNA, and their parent cell line 143B.TK¯. The significant decrease in the global ubiquitination pattern in ρ^0^ cells can be explained by the lack of main ROS generators localized within the oxidative phosphorylation system, namely complex I and III, which reduce the oxidative damage of proteins to a minimal level [47].

This combination of DUB and GSH inhibitors has not been applied to RCC cell lines yet but would present a valuable new strategy to trigger proteotoxic stress as a potential beneficial treatment in RCC cell lines and animal models.

### 4.5. The Role of GSH Metabolism in the Immune Microenvironment of the Tumor

Traditional immunotherapies using interleukin-2 or interferon-alfa on metastatic RCC have presented limited efficacy and highly toxic side effects [117,118]. In recent years, a new generation of immunotherapy utilizing a novel strategy to block immune checkpoints has shown promising efficacy and manageable toxicity and has emerged as a new milestone for RCC treatment [119]. Currently, the approved immune checkpoint inhibitors for RCC are ipilimumab (a cytotoxic T lymphocyte-associated protein 4 [CTLA-4] inhibitor) [120], the programmed cell death 1 (PD-1)-specific antibodies nivolumab [121] and pembrolizumab, and the programmed death-ligand 1 (PD-L1) antibody avelumab.

A main metabolic feature of RCC is the reprogramming of the main metabolic pathways, which helps cancer cells adapt to and simultaneously shape the tumor microenvironment. Immune cells utilize different metabolic programs for their differentiation and effective functions. These immune–metabolic pathways can be modified or “highjacked” in the tumor microenvironment and thus affect the normal functions of immune cells, e.g., by infiltrating tumor tissues and presenting tumor-associated antigens to T-cells [122]. Therefore, a huge effort has been put into the development of immunotherapies to take advantage of the complex crosstalk between immune cells and the tumor [123].

How does GSH metabolism influence this crosstalk in RCC? Though there is sparse literature on this topic, it has been shown that GSH metabolism can influence the immune microenvironment in cancer at least in the following two aspects. First, glutamine is a crucial nutrient for the effector function of T-cells. Glutamine deprivation or its transporter deficiency blocks the differentiation of T-helper 1 and 17 cells [124,125]. Similarly, the proliferation and differentiation of B-cells also requires glutamine [126]. As glutamine addiction is one of the main features of RCC, glutamine may be a limiting nutrient factor in the tumor microenvironment, and thus a lack of glutamine can induce immunosuppression. Second, T-cell stimulation activates the cystine–glutamate antiporter xCT and leads to increased uptake of cystine and subsequent GSH synthesis [122]. Reduced GSH levels in antigen-presenting cells have been shown to influence antigen processing and presentation as well as T-cell differentiation into T-helper 1 or 2 phenotypes [127]. Furthermore, GSH can bind to anticancer drugs, and these conjugates can be effluxed out of the cell via multiple resistance-associated protein transporters, which are the underlying reasons for therapeutic resistance in some cancers [128]. All the points discussed above impressively show how tumor cells compete with immune cells for GSH-related nutrients in the tumor microenvironment, but more research on the immune response in RCC is needed to exploit these mechanisms for new therapy development.

## 5. Conclusions

Altered GSH metabolism contributes significantly to the development and progression of all renal malignancies but could, at the same time, be the key to potential therapies. All RCCs have a reduced oxidative phosphorylation capacity in common. The dysregulated respiratory chain is the main source of electron leakage, resulting in excessive ROS. Raised oxidative stress levels in RCC are counteracted by tremendously increased GSH levels and thereby potentially prevent immune reactions, apoptosis, or other forms of cell death as a strategy to foster the survival of the malignancy. Many applied chemotherapeutics initiate an additional production of ROS as one potent mechanism to eradicate RCC, frequently accompanied by the supplementation of antioxidants in the past. Not surprisingly, targeting ROS by antioxidants and the simultaneous generation of ROS by chemotherapeutics has led to mixed results in the treatability of RCC [14]. New therapeutic ways exploit sensitivity toward inhibitors of the GSH metabolism, such as xCT, glutaminase, and GCL. However, the inhibition of just one altered pathway to cure RCC turned out to be not successful either, as cells are fitted by a very flexible system to compensate for the impairment of one pathway or mechanism. New studies have shown that a combinatory therapy targeting two independent pathways and one involved in ROS metabolism is key to improving the survival rate and eventually curing RCC. Although the main role of GSH and other antioxidants is to scavenge intracellular ROS to maintain an overall healthy pro- and antioxidant exposure status in cells, GSH can also function as a signaling molecule or as a donor of the post-translational modification S-glutathionylation to regulate protein bioactivity, which was not discussed in this review. These diverse functions of GSH, a molecule that was identified more than 100 years ago, still need to be further investigated for better understanding of the underlying disease mechanisms in cancer. This might facilitate the development of GSH-related modulators with improved therapeutic efficiencies in the future.

## Figures and Tables

**Table 1:** Summary of the three renal cell carcinoma (RCC) subtypes, incidences, main mutations, and GSH regulation.

RCC Subtypes	Clear Cell	Papillary	Chromophobe
Incidence	75%	15%	5%
Main mutations	*VHL*	*MET, FH*	*TP53, PTEN*
Metabolites	GSH, GSSG increased	GSH, GSSG increased	GSH, GSSG increased
GSH regulation	1. GCL protein abundance increases; 2. Increased serum GGT as a marker for metastatic ccRCC; 3. GLS1, glutamine importers, and cysteine antiporter xCT enhance to favor GSH synthesis; 4. Increased PPP flux to produce NADPH for GSH conversion.	1. *FH* mutation causes HIF stabilization; 2. *FH* mutation activates NRF2–ARE pathway, leading to increased GSH synthesis and enhanced expression of antioxidant proteins.	Loss of GGT1 increases sensitivity to oxidative stress in chRCC cells.

ccRCC: clear cell RCC; chRCC: chromophobe RCC; pRCC: papillary RCC; *VHL*: von Hippel-Lindau; *MET*: proto-oncogene c-Met; *FH*: fumarate hydrotase; *TP53*: tumor antigen p53; *PTEN*: phosphatase and tensin homolog; PPP: pentose phosphate pathway; HIF: hypoxia-inducible factor; NRF2: nuclear factor erythroid 2-related factor 2; ARE: antioxidant response element; GGT1: γ-glutamyl transferase 1.

## Abbreviations

968	5-[3-bromo-4-(dimethylamino)phenyl]-2,3,5,6-tetrahydro-2,2-dimethyl-benzo[A]phenanth-ridin-4(1H)-one
ALDOB	Aldolase B
ARE	Antioxidant response element
BPTES	Bis-2-(5-phenylacetamido-1,2,4-thiadiazol-2-Yl)ethyl sulfide
BRAF	Proto-oncogene B-RAF
BSO	Buthionine sulfoximine
ccRCC	Clear cell renal cell carcinoma
CDKN2A	Cyclin-dependent kinase inhibitor 2A
CDKN2B	Cyclin-dependent kinase inhibitor 2B
chRCC	Chromophobe renal cell carcinoma
CRAF	Proto-oncogene C-RAF
CTLA-4	Cytotoxic T lymphocyte-associated protein 4
CUL3	Cullin 3
DCR	Disease control rate
DON	6-diazo-5-oxo-l-norleucine
DUB	Deubiquitinating enzyme
EGFR	Epidermal growth factor receptor
EGLN	Egl-nine homolog
FAK	Focal adhesion kinase
FBP	Fructose 1,6-bisphosphate
FBP1	Fructose 1,6-bisphosphatase 1
FH	Fumarate hydratase
FLT3	Fms-like tyrosine kinase 3
G6PD	Glucose-6-phosphate dehydrogenase
GCL	Glutamate cysteine ligase
GCLC	Glutamate cysteine ligase catalytic subunit
GCLM	Glutamate cysteine ligase modulatory subunit
GGT	γ-glutamyl transferase
GGT1	γ-glutamyl transferase 1
GLS1	Glutaminase 1
GLS2	Glutaminase 2
GLUD	Glutamate dehydrogenase
GLUT1	Glucose transporter type 1
GPX	Glutathione peroxidase
GR	Glutathione reductase
GSH	Glutathione reduced form
GSS	Glutathione synthetase
GSSG	Glutathione oxidized form
GST	Glutathione S-transferase
HGF	Hepatocyte growth factor
HIF	Hypoxia inducible factor
KEAP1	Kelch-like ECH-associated protein 1
KIT	Tyrosine-protein kinase kit
MET	Proto-oncogene c-Met
MYC	MYC proto-oncogene
NADPH	Nicotinamide adenine dinucleotide phosphate
NOX4	NADPH oxidase 4
NRAS	NRAS proto-oncogene, GTPase
NRF2	Nuclear factor erythroid 2-related factor 2
ODD	Oxygen-dependent degradation
PC	Pyruvate carboxylase
PD-1	Programmed cell death 1
PD-L1	Programmed cell death-ligand 1
PDGFR	Platelet-derived growth factor receptor
PPP	Pentose phosphate pathway
pRCC	Papillary renal cell carcinoma
R5P	Ribose 5-phosphate
RCC	Renal cell carcinoma
RET	RET proto-oncogene
ROS	Reactive oxygen species
RTK	Receptor tyrosine kinase
SAH	S-adenosyl homocysteine
SAM	S-adenosyl methionine
SETD2	Set domain-containing 2, histone lysine methyltransferase
SLC38A1	Solute carrier family 38 member 1
SLC7A11	Solute carrier family 7 member 11
TERT	Telomerase reverse transcriptase
TFE3	Transcription factor E3
TSC1	Tuberous sclerosis 1 protein
TSC2	Tuberous sclerosis 2 protein
VDAC1	Voltage-dependent anion channel 1
VDAC2	Voltage-dependent anion channel 2
VDAC3	Voltage-dependent anion channel 3
VEGF	Vascular endothelial growth factor
VEGFR	Vascular endothelial growth factor receptor
VHL	von Hippel-Lindau

## References

[1] Kim J., Kim J., Bae J.S. (2016). ROS homeostasis and metabolism: A critical liaison for cancer therapy. Exp. Mol. Med..

[2] Meister A. (1988). On the discovery of glutathione. Trends. Biochem. Sci..

[3] Kaplowitz N., Aw T.Y., Ookhtens M. (1985). The regulation of hepatic glutathione. Annu. Rev. Pharmacol. Toxicol..

[4] Meister A., Anderson M.E. (1983). Glutathione. Annu. Rev. Biochem..

[5] Lu S.C. (2009). Regulation of glutathione synthesis. Mol. Asp. Med..

[6] Cho E.S., Johnson N., Snider B.C. (1984). Tissue glutathione as a cyst(e)ine reservoir during cystine depletion in growing rats. J. Nutr..

[7] Sipos K., Lange H., Fekete Z., Ullmann P., Lill R., Kispal G. (2002). Maturation of cytosolic iron-sulfur proteins requires glutathione. J. Biol. Chem..

[8] Awasthi Y.C., Misra G., Rassin D.K., Srivastava S.K. (1983). Detoxification of xenobiotics by glutathione S-transferases in erythrocytes: The transport of the conjugate of glutathione and 1-chloro-2,4-dinitrobenzene. Br. J. Haematol..

[9] Duan J., Kodali V.K., Gaffrey M.J., Guo J., Chu R.K., Camp D.G., Smith R.D., Thrall B.D., Qian W.J. (2016). Quantitative Profiling of Protein S-Glutathionylation Reveals Redox-Dependent Regulation of Macrophage Function during Nanoparticle-Induced Oxidative Stress. ACS Nano.

[10] Zhang X., Liu P., Zhang C., Chiewchengchol D., Zhao F., Yu H., Li J., Kambara H., Luo K.Y., Venkataraman A. (2017). Positive Regulation of Interleukin-1beta Bioactivity by Physiological ROS-Mediated Cysteine S-Glutathionylation. Cell. Rep..

[11] Ren X., Zou L., Zhang X., Branco V., Wang J., Carvalho C., Holmgren A., Lu J. (2017). Redox Signaling Mediated by Thioredoxin and Glutathione Systems in the Central Nervous System. Antioxid. Redox Signal..

[12] Hussain S.P., Hofseth L.J., Harris C.C. (2003). Radical causes of cancer. Nat. Rev. Cancer.

[13] Hatem E., El Banna N., Huang M.E. (2017). Multifaceted Roles of Glutathione and Glutathione-Based Systems in Carcinogenesis and Anticancer Drug Resistance. Antioxid. Redox Signal..

[14] Thyagarajan A., Sahu R.P. (2018). Potential Contributions of Antioxidants to Cancer Therapy: Immunomodulation and Radiosensitization. Integr. Cancer.

[15] Siegel R.L., Miller K.D., Jemal A. (2019). Cancer statistics, 2019. CA. Cancer J. Clin..

[16] Bray F., Ferlay J., Soerjomataram I., Siegel R.L., Torre L.A., Jemal A. (2018). Global cancer statistics 2018: GLOBOCAN estimates of incidence and mortality worldwide for 36 cancers in 185 countries. CA Cancer J. Clin..

[17] Sato Y., Yoshizato T., Shiraishi Y., Maekawa S., Okuno Y., Kamura T., Shimamura T., Sato-Otsubo A., Nagae G., Suzuki H. (2013). Integrated molecular analysis of clear-cell renal cell carcinoma. Nat. Genet..

[18] Linehan W.M., Spellman P.T., Ricketts C.J., Creighton C.J., Fei S.S., Davis C., Wheeler D.A., Murray B.A., Schmidt L., Cancer Genome Atlas Research Network (2016). Comprehensive Molecular Characterization of Papillary Renal-Cell Carcinoma. N. Engl. J. Med..

[19] Davis C.F., Ricketts C.J., Wang M., Yang L., Cherniack A.D., Shen H., Buhay C., Kang H., Kim S.C., Fahey C.C. (2014). The somatic genomic landscape of chromophobe renal cell carcinoma. Cancer Cell.

[20] Ricketts C.J., De Cubas A.A., Fan H., Smith C.C., Lang M., Reznik E., Bowlby R., Gibb E.A., Akbani R., Beroukhim R. (2018). The Cancer Genome Atlas Comprehensive Molecular Characterization of Renal Cell Carcinoma. Cell. Rep..

[21] Hoadley K.A., Yau C., Hinoue T., Wolf D.M., Lazar A.J., Drill E., Shen R., Taylor A.M., Cherniack A.D., Thorsson V. (2018). Cell-of-Origin Patterns Dominate the Molecular Classification of 10,000 Tumors from 33 Types of Cancer. Cell.

[22] Moch H., Cubilla A.L., Humphrey P.A., Reuter V.E., Ulbright T.M. (2016). The 2016 WHO Classification of Tumours of the Urinary System and Male Genital Organs-Part A: Renal, Penile, and Testicular Tumours. Eur. Urol..

[23] Chin A.I., Lam J.S., Figlin R.A., Belldegrun A.S. (2006). Surveillance strategies for renal cell carcinoma patients following nephrectomy. Rev. Urol..

[24] Motzer R.J., Bacik J., Mazumdar M. (2004). Prognostic factors for survival of patients with stage IV renal cell carcinoma: Memorial sloan-kettering cancer center experience. Clin. Cancer Res..

[25] Delahunt B., Srigley J.R., Montironi R., Egevad L. (2014). Advances in renal neoplasia: Recommendations from the 2012 International Society of Urological Pathology Consensus Conference. Urology.

[26] Cancer Genome Atlas Research Network (2013). Comprehensive molecular characterization of clear cell renal cell carcinoma. Nature.

[27] Latif F., Tory K., Gnarra J., Yao M., Duh F.M., Orcutt M.L., Stackhouse T., Kuzmin I., Modi W., Geil L. (1993). Identification of the von Hippel-Lindau disease tumor suppressor gene. Science.

[28] Jaakkola P., Mole D.R., Tian Y.M., Wilson M.I., Gielbert J., Gaskell S.J., von Kriegsheim A., Hebestreit H.F., Mukherji M., Schofield C.J. (2001). Targeting of HIF-alpha to the von Hippel-Lindau ubiquitylation complex by O2-regulated prolyl hydroxylation. Science.

[29] Ivan M., Kondo K., Yang H., Kim W., Valiando J., Ohh M., Salic A., Asara J.M., Lane W.S., Kaelin W.G. (2001). HIFalpha targeted for VHL-mediated destruction by proline hydroxylation: Implications for O_2_ sensing. Science.

[30] Gossage L., Eisen T., Maher E.R. (2015). VHL, the story of a tumour suppressor gene. Nat. Rev. Cancer.

[31] Riazalhosseini Y., Lathrop M. (2016). Precision medicine from the renal cancer genome. Nat. Rev. Nephrol..

[32] Wettersten H.I., Aboud O.A., Lara P.N., Weiss R.H. (2017). Metabolic reprogramming in clear cell renal cell carcinoma. Nat. Rev. Nephrol..

[33] Posadas E.M., Limvorasak S., Figlin R.A. (2017). Targeted therapies for renal cell carcinoma. Nat. Rev. Nephrol..

[34] Li B., Qiu B., Lee D.S., Walton Z.E., Ochocki J.D., Mathew L.K., Mancuso A., Gade T.P., Keith B., Nissim I. (2014). Fructose-1,6-bisphosphatase opposes renal carcinoma progression. Nature.

[35] Guo T., Kouvonen P., Koh C.C., Gillet L.C., Wolski W.E., Rost H.L., Rosenberger G., Collins B.C., Blum L.C., Gillessen S. (2015). Rapid mass spectrometric conversion of tissue biopsy samples into permanent quantitative digital proteome maps. Nat. Med..

[36] Hakimi A.A., Reznik E., Lee C.H., Creighton C.J., Brannon A.R., Luna A., Aksoy B.A., Liu E.M., Shen R., Lee W. (2016). An Integrated Metabolic Atlas of Clear Cell Renal Cell Carcinoma. Cancer Cell.

[37] Steffens S., Janssen M., Roos F.C., Becker F., Schumacher S., Seidel C., Wegener G., Thüroff J.W., Hofmann R., Stöckle M. (2012). Incidence and long-term prognosis of papillary compared to clear cell renal cell carcinoma—A multicentre study. Eur. J. Cancer.

[38] Delahunt B., Eble J.N. (1997). Papillary renal cell carcinoma: A clinicopathologic and immunohistochemical study of 105 tumors. Mod. Pathol..

[39] Pignot G., Elie C., Conquy S., Vieillefond A., Flam T., Zerbib M., Debre B., Amsellem-Ouazana D. (2007). Survival analysis of 130 patients with papillary renal cell carcinoma: Prognostic utility of type 1 and type 2 subclassification. Urology.

[40] Pal S.K., Ali S.M., Yakirevich E., Geynisman D.M., Karam J.A., Elvin J.A., Frampton G.M., Huang X., Lin D.I., Rosenzweig M. (2018). Characterization of Clinical Cases of Advanced Papillary Renal Cell Carcinoma via Comprehensive Genomic Profiling. Eur. Urol..

[41] Fay A.P., Signoretti S., Choueiri T.K. (2014). MET as a target in papillary renal cell carcinoma. Clin. Cancer Res..

[42] Li S., Shuch B.M., Gerstein M.B. (2017). Whole-genome analysis of papillary kidney cancer finds significant noncoding alterations. PLoS Genet..

[43] Tomlinson I., Alam N., Rowan A., Barclay E., Jaeger E., Kelsell D., Leigh I., Gorman P., Lamlum H., Rahman S. (2002). Multiple Leiomyoma Consortium: Germline mutations in FH predispose to dominantly inherited uterine fibroids, skin leiomyomata and papillary renal cell cancer. Nat. Genet..

[44] Pollard P.J., Briere J.J., Alam N.A., Barwell J., Barclay E., Wortham N.C., Hunt T., Mitchell M., Olpin S., Moat S.J. (2005). Accumulation of Krebs cycle intermediates and over-expression of HIF1alpha in tumours which result from germline FH and SDH mutations. Hum. Mol. Genet..

[45] Sullivan L.B., Martinez-Garcia E., Nguyen H., Mullen A.R., Dufour E., Sudarshan S., Licht J.D., Deberardinis R.J., Chandel N.S. (2013). The proto-oncometabolite fumarate binds glutathione to amplify ROS-dependent signaling. Mol. Cell.

[46] Alahmad A., Paffrath V., Clima R., Busch J.F., Rabien A., Kilic E., Villegas S., Timmermann B., Attimonelli M., Jung K. (2019). Papillary renal cell carcinomas rewire glutathione metabolism and are deficient in anabolic glucose synthesis. bioRxiv.

[47] Aretz I., Hardt C., Wittig I., Meierhofer D. (2016). An Impaired Respiratory Electron Chain Triggers Down-regulation of the Energy Metabolism and De-ubiquitination of Solute Carrier Amino Acid Transporters. Mol. Cell. Proteom. MCP.

[48] Hu H., Nan J., Sun Y., Zhu D., Xiao C., Wang Y., Zhu L., Wu Y., Zhao J., Wu R. (2017). Electron leak from NDUFA13 within mitochondrial complex I attenuates ischemia-reperfusion injury via dimerized STAT3. Proc. Natl. Acad. Sci. USA.

[49] Jastroch M., Divakaruni A.S., Mookerjee S., Treberg J.R., Brand M.D. (2010). Mitochondrial proton and electron leaks. Essays Biochem..

[50] Hirst J., Roessler M.M. (2016). Energy conversion, redox catalysis and generation of reactive oxygen species by respiratory complex I. Biochim. Biophys. Acta.

[51] Reichart G., Mayer J., Zehm C., Kirschstein T., Tokay T., Lange F., Baltrusch S., Tiedge M., Fuellen G., Ibrahim S. (2019). Mitochondrial complex IV mutation increases reactive oxygen species production and reduces lifespan in aged mice. Acta Physiol. (Oxf.).

[52] Thoenes W., Storkel S., Rumpelt H.J. (1985). Human chromophobe cell renal carcinoma. Virchows Arch. B Cell Pathol. Incl. Mol. Pathol..

[53] Yusenko M.V. (2010). Molecular pathology of chromophobe renal cell carcinoma: A review. Int. J. Urol..

[54] Haake S.M., Weyandt J.D., Rathmell W.K. (2016). Insights into the Genetic Basis of the Renal Cell Carcinomas from The Cancer Genome Atlas. Mol. Cancer Res..

[55] Brunelli M., Eble J.N., Zhang S., Martignoni G., Delahunt B., Cheng L. (2005). Eosinophilic and classic chromophobe renal cell carcinomas have similar frequent losses of multiple chromosomes from among chromosomes 1, 2, 6, 10, and 17, and this pattern of genetic abnormality is not present in renal oncocytoma. Mod. Pathol. Off. J. United States Can. Acad. Pathol. Inc..

[56] Casuscelli J., Weinhold N., Gundem G., Wang L., Zabor E.C., Drill E., Wang P.I., Nanjangud G.J., Redzematovic A., Nargund A.M. (2017). Genomic landscape and evolution of metastatic chromophobe renal cell carcinoma. JCI Insight.

[57] Xiao Y., Clima R., Busch J.F., Rabien A., Kilic E., Villegas S., Türkmen S., Timmermann B., Attimonelli M., Jung K. (2019). Metabolic reprogramming and elevation of glutathione in chromophobe renal cell carcinomas. bioRxiv..

[58] Jinzaki M., Tanimoto A., Mukai M., Ikeda E., Kobayashi S., Yuasa Y., Narimatsu Y., Murai M. (2000). Double-phase helical CT of small renal parenchymal neoplasms: Correlation with pathologic findings and tumor angiogenesis. J. Comput. Assist. Tomogr..

[59] Nakajima R., Nozaki S., Kondo T., Nagashima Y., Abe K., Sakai S. (2017). Evaluation of renal cell carcinoma histological subtype and fuhrman grade using (18)F-fluorodeoxyglucose-positron emission tomography/computed tomography. Eur. Radiol..

[60] Priolo C., Khabibullin D., Reznik E., Filippakis H., Ogorek B., Kavanagh T.R., Nijmeh J., Herbert Z.T., Asara J.M., Kwiatkowski D.J. (2018). Impairment of gamma-glutamyl transferase 1 activity in the metabolic pathogenesis of chromophobe renal cell carcinoma. Proc. Natl. Acad. Sci. USA.

[61] Kurschner G., Zhang Q., Clima R., Xiao Y., Busch J.F., Kilic E., Jung K., Berndt N., Bulik S., Holzhutter H.G. (2017). Renal oncocytoma characterized by the defective complex I of the respiratory chain boosts the synthesis of the ROS scavenger glutathione. Oncotarget.

[62] Gopal R.K., Calvo S.E., Shih A.R., Chaves F.L., McGuone D., Mick E., Pierce K.A., Li Y., Garofalo A., Van Allen E.M. (2018). Early loss of mitochondrial complex I and rewiring of glutathione metabolism in renal oncocytoma. Proc. Natl. Acad. Sci. USA.

[63] Orlowski M., Meister A. (1970). The gamma-glutamyl cycle: A possible transport system for amino acids. Proc. Natl. Acad. Sci. USA.

[64] Franklin C.C., Backos D.S., Mohar I., White C.C., Forman H.J., Kavanagh T.J. (2009). Structure, function, and post-translational regulation of the catalytic and modifier subunits of glutamate cysteine ligase. Mol. Asp. Med..

[65] Li M., Zhang Z., Yuan J., Zhang Y., Jin X. (2014). Altered glutamate cysteine ligase expression and activity in renal cell carcinoma. Biomed. Rep..

[66] Miess H., Dankworth B., Gouw A.M., Rosenfeldt M., Schmitz W., Jiang M., Saunders B., Howell M., Downward J., Felsher D.W. (2018). The glutathione redox system is essential to prevent ferroptosis caused by impaired lipid metabolism in clear cell renal cell carcinoma. Oncogene.

[67] Terzyan S.S., Burgett A.W., Heroux A., Smith C.A., Mooers B.H., Hanigan M.H. (2015). Human Gamma-Glutamyl Transpeptidase 1: Structures Of The Free Enzyme, Inhibitor-Bound Tetrahedral Transition States, And Glutamate-Bound Enzyme Reveal Novel Movement Within The Active Site During Catalysis. J. Biol. Chem..

[68] Simic T., Dragicevic D., Savic-Radojevic A., Cimbaljevic S., Tulic C., Mimic-Oka J. (2007). Serum gamma glutamyl-transferase is a sensitive but unspecific marker of metastatic renal cell carcinoma. Int. J. Urol..

[69] Hofbauer S.L., Stangl K.I., de Martino M., Lucca I., Haitel A., Shariat S.F., Klatte T. (2014). Pretherapeutic gamma-glutamyltransferase is an independent prognostic factor for patients with renal cell carcinoma. Br. J. Cancer.

[70] Fu Q., Xu L., Wang Y., Jiang Q., Liu Z., Zhang J., Zhou Q., Zeng H., Tong S., Wang T. (2019). Tumor-associated Macrophage-derived Interleukin-23 Interlinks Kidney Cancer Glutamine Addiction with Immune Evasion. Eur. Urol..

[71] Abu Aboud O., Habib S.L., Trott J., Stewart B., Liang S., Chaudhari A.J., Sutcliffe J., Weiss R.H. (2017). Glutamine Addiction in Kidney Cancer Suppresses Oxidative Stress and Can Be Exploited for Real-Time Imaging. Cancer Res..

[72] Hoerner C.R., Chen V.J., Fan A.C. (2019). The ‘Achilles Heel’ of Metabolism in Renal Cell Carcinoma: Glutaminase Inhibition as a Rational Treatment Strategy. Kidney Cancer.

[73] Broer A., Rahimi F., Broer S. (2016). Deletion of Amino Acid Transporter ASCT2 (SLC1A5) Reveals an Essential Role for Transporters SNAT1 (SLC38A1) and SNAT2 (SLC38A2) to Sustain Glutaminolysis in Cancer Cells. J. Biol. Chem..

[74] Bailey S.T., Smith A.M., Kardos J., Wobker S.E., Wilson H.L., Krishnan B., Saito R., Lee H.J., Zhang J., Eaton S.C. (2017). MYC activation cooperates with Vhl and Ink4a/Arf loss to induce clear cell renal cell carcinoma. Nat. Commun..

[75] Tang S.W., Chang W.H., Su Y.C., Chen Y.C., Lai Y.H., Wu P.T., Hsu C.I., Lin W.C., Lai M.K., Lin J.Y. (2009). MYC pathway is activated in clear cell renal cell carcinoma and essential for proliferation of clear cell renal cell carcinoma cells. Cancer Lett..

[76] Son J., Lyssiotis C.A., Ying H., Wang X., Hua S., Ligorio M., Perera R.M., Ferrone C.R., Mullarky E., Shyh-Chang N. (2013). Glutamine supports pancreatic cancer growth through a KRAS-regulated metabolic pathway. Nature.

[77] Kredich N.M. (2008). Biosynthesis of Cysteine. EcoSal Plus.

[78] Bansal A., Simon M.C. (2018). Glutathione metabolism in cancer progression and treatment resistance. J. Cell Biol..

[79] Lucarelli G., Galleggiante V., Rutigliano M., Sanguedolce F., Cagiano S., Bufo P., Lastilla G., Maiorano E., Ribatti D., Giglio A. (2015). Metabolomic profile of glycolysis and the pentose phosphate pathway identifies the central role of glucose-6-phosphate dehydrogenase in clear cell-renal cell carcinoma. Oncotarget.

[80] Wang J., Wu Q., Qiu J. (2019). Accumulation of fructose 1,6-bisphosphate protects clear cell renal cell carcinoma from oxidative stress. Lab. Investig..

[81] Mullen A.R., Wheaton W.W., Jin E.S., Chen P.H., Sullivan L.B., Cheng T., Yang Y., Linehan W.M., Chandel N.S., DeBerardinis R.J. (2011). Reductive carboxylation supports growth in tumour cells with defective mitochondria. Nature.

[82] Ooi A., Wong J.C., Petillo D., Roossien D., Perrier-Trudova V., Whitten D., Min B.W., Tan M.H., Zhang Z., Yang X.J. (2011). An antioxidant response phenotype shared between hereditary and sporadic type 2 papillary renal cell carcinoma. Cancer Cell.

[83] Adam J., Hatipoglu E., O’Flaherty L., Ternette N., Sahgal N., Lockstone H., Baban D., Nye E., Stamp G.W., Wolhuter K. (2011). Renal cyst formation in Fh1-deficient mice is independent of the Hif/Phd pathway: Roles for fumarate in KEAP1 succination and Nrf2 signaling. Cancer Cell.

[84] Jaramillo M.C., Zhang D.D. (2013). The emerging role of the Nrf2-Keap1 signaling pathway in cancer. Genes Dev..

[85] Kansanen E., Kuosmanen S.M., Leinonen H., Levonen A.L. (2013). The Keap1-Nrf2 pathway: Mechanisms of activation and dysregulation in cancer. Redox Biol..

[86] Tong K.I., Katoh Y., Kusunoki H., Itoh K., Tanaka T., Yamamoto M. (2006). Keap1 recruits Neh2 through binding to ETGE and DLG motifs: Characterization of the two-site molecular recognition model. Mol. Cell Biol..

[87] Taguchi K., Yamamoto M. (2017). The KEAP1-NRF2 System in Cancer. Front. Oncol..

[88] Jin L., Li D., Alesi G.N., Fan J., Kang H.B., Lu Z., Boggon T.J., Jin P., Yi H., Wright E.R. (2015). Glutamate dehydrogenase 1 signals through antioxidant glutathione peroxidase 1 to regulate redox homeostasis and tumor growth. Cancer Cell.

[89] Cairns P. (2010). Renal cell carcinoma. Cancer Biomark.

[90] Shojaei F., Lee J.H., Simmons B.H., Wong A., Esparza C.O., Plumlee P.A., Feng J., Stewart A.E., Hu-Lowe D.D., Christensen J.G. (2010). HGF/c-Met acts as an alternative angiogenic pathway in sunitinib-resistant tumors. Cancer Res..

[91] Bielecka Z.F., Czarnecka A.M., Solarek W., Kornakiewicz A., Szczylik C. (2014). Mechanisms of Acquired Resistance to Tyrosine Kinase Inhibitors in Clear - Cell Renal Cell Carcinoma (ccRCC). Curr. Signal. Transduct..

[92] Harris I.S., Endress J.E., Coloff J.L., Selfors L.M., McBrayer S.K., Rosenbluth J.M., Takahashi N., Dhakal S., Koduri V., Oser M.G. (2019). Deubiquitinases Maintain Protein Homeostasis and Survival of Cancer Cells upon Glutathione Depletion. Cell Metab..

[93] Lewerenz J., Hewett S.J., Huang Y., Lambros M., Gout P.W., Kalivas P.W., Massie A., Smolders I., Methner A., Pergande M. (2013). The cystine/glutamate antiporter system xc− in health and disease: From molecular mechanisms to novel therapeutic opportunities. Antioxid. Redox Signal..

[94] Dixon S.J., Patel D.N., Welsch M., Skouta R., Lee E.D., Hayano M., Thomas A.G., Gleason C.E., Tatonetti N.P., Slusher B.S. (2014). Pharmacological inhibition of cystine-glutamate exchange induces endoplasmic reticulum stress and ferroptosis. eLife.

[95] Keating G.M. (2017). Sorafenib: A Review in Hepatocellular Carcinoma. Target. Oncol..

[96] Sehm T., Rauh M., Wiendieck K., Buchfelder M., Eyupoglu I.Y., Savaskan N.E. (2016). Temozolomide toxicity operates in a xCT/SLC7a11 dependent manner and is fostered by ferroptosis. Oncotarget.

[97] Roh J.L., Kim E.H., Jang H., Shin D. (2017). Aspirin plus sorafenib potentiates cisplatin cytotoxicity in resistant head and neck cancer cells through xCT inhibition. Free Radic. Biol. Med..

[98] Dixon S.J., Lemberg K.M., Lamprecht M.R., Skouta R., Zaitsev E.M., Gleason C.E., Patel D.N., Bauer A.J., Cantley A.M., Yang W.S. (2012). Ferroptosis: An iron-dependent form of nonapoptotic cell death. Cell.

[99] Yagoda N., von Rechenberg M., Zaganjor E., Bauer A.J., Yang W.S., Fridman D.J., Wolpaw A.J., Smukste I., Peltier J.M., Boniface J.J. (2007). RAS–RAF–MEK-dependent oxidative cell death involving voltage-dependent anion channels. Nature.

[100] Tang X., Wu J., Ding C.K., Lu M., Keenan M.M., Lin C.C., Lin C.A., Wang C.C., George D., Hsu D.S. (2016). Cystine Deprivation Triggers Programmed Necrosis in VHL-Deficient Renal Cell Carcinomas. Cancer Res..

[101] Momcilovic M., Bailey S.T., Lee J.T., Fishbein M.C., Magyar C., Braas D., Graeber T., Jackson N.J., Czernin J., Emberley E. (2017). Targeted Inhibition of EGFR and Glutaminase Induces Metabolic Crisis in EGFR Mutant Lung Cancer. Cell Rep..

[102] Emberley E., Bennett M., Chen J., Gross M., Huang T., Li W., Mackinnon A., Pan A., Rodriguez M., Steggerda S. (2016). CB-839, a selective glutaminase inhibitor, has anti-tumor activity in renal cell carcinoma and synergizes with everolimus and receptor tyrosine kinase inhibitors. Eur. J. Cancer.

[103] MacKinnon A.L., Bennett M., Gross M., Janes J., Li W.Q., Rodriquez M., Wang T., Zhang W., Parlati F. (2015). Metabolomic, Proteomic and Genomic Profiling Identifies Biomarakers of Sensitivity to Glutaminase Inhibitor CB-839 in Multiple Myeloma. Blood.

[104] Meric-Bernstam F., Tannir N.M., Mier J.W., DeMichele A., Telli M.L., Fan A.C., Munster P.N., Carvajal R.D., Orford K.W., Bennett M.K. (2016). Phase 1 study of CB-839, a small molecule inhibitor of glutaminase (GLS), alone and in combination with everolimus (E) in patients (pts) with renal cell cancer (RCC). J. Clin. Oncol..

[105] Okazaki A., Gameiro P.A., Christodoulou D., Laviollette L., Schneider M., Chaves F., Stemmer-Rachamimov A., Yazinski S.A., Lee R., Stephanopoulos G. (2017). Glutaminase and poly(ADP-ribose) polymerase inhibitors suppress pyrimidine synthesis and VHL-deficient renal cancers. J. Clin. Investig..

[106] Song M., Kim S.H., Im C.Y., Hwang H.J. (2018). Recent Development of Small Molecule Glutaminase Inhibitors. Curr. Top. Med. Chem..

[107] Bailey H.H. (1998). l-S,R-buthionine sulfoximine: Historical development and clinical issues. Chem. Biol. Interact..

[108] Griffith O.W., Meister A. (1979). Potent and specific inhibition of glutathione synthesis by buthionine sulfoximine (Sn-butyl homocysteine sulfoximine). J. Biol. Chem..

[109] Tagde A., Singh H., Kang M.H., Reynolds C.P. (2014). The glutathione synthesis inhibitor buthionine sulfoximine synergistically enhanced melphalan activity against preclinical models of multiple myeloma. Blood Cancer J..

[110] Anderson C.P., Matthay K.K., Perentesis J.P., Neglia J.P., Bailey H.H., Villablanca J.G., Groshen S., Hasenauer B., Maris J.M., Seeger R.C. (2015). Pilot study of intravenous melphalan combined with continuous infusion l-*S*,*R*-buthionine sulfoximine for children with recurrent neuroblastoma. Pediatr. Blood Cancer.

[111] Villablanca J.G., Volchenboum S.L., Cho H., Kang M.H., Cohn S.L., Anderson C.P., Marachelian A., Groshen S., Tsao-Wei D., Matthay K.K. (2016). A Phase I New Approaches to Neuroblastoma Therapy Study of Buthionine Sulfoximine and Melphalan With Autologous Stem Cells for Recurrent/Refractory High-Risk Neuroblastoma. Pediatr. Blood Cancer.

[112] Zhang L., Bhasin M., Schor-Bardach R., Wang X., Collins M.P., Panka D., Putheti P., Signoretti S., Alsop D.C., Libermann T. (2011). Resistance of renal cell carcinoma to sorafenib is mediated by potentially reversible gene expression. PLoS ONE.

[113] Panka D., Kumar M., Schor-Bardach R., Zhang L., Atkins M., Libermann T., Goldberg N., Bhatt R., Mier J. (2008). Mechanism of acquired resistance to sorafenib in RCC. Cancer Res..

[114] Nikinmaa M., Pursiheimo S., Soitamo A.J. (2004). Redox state regulates HIF-1alpha and its DNA binding and phosphorylation in salmonid cells. J. Cell Sci..

[115] Chen H., Shi H. (2008). A reducing environment stabilizes HIF-2alpha in SH-SY5Y cells under hypoxic conditions. FEBS Lett..

[116] Jin W.-s., Kong Z.-l., Shen Z.-f., Jin Y.-z., Zhang W.-k., Chen G.-f. (2011). Regulation of hypoxia inducible factor-1α expression by the alteration of redox status in HepG2 cells. J. Exp. Clin. Cancer Res. CR.

[117] Fyfe G., Fisher R.I., Rosenberg S.A., Sznol M., Parkinson D.R., Louie A.C. (1995). Results of treatment of 255 patients with metastatic renal cell carcinoma who received high-dose recombinant interleukin-2 therapy. J. Clin. Oncol. Off. J. Am. Soc. Clin. Oncol..

[118] Negrier S., Escudier B., Lasset C., Douillard J.Y., Savary J., Chevreau C., Ravaud A., Mercatello A., Peny J., Mousseau M. (1998). Recombinant human interleukin-2, recombinant human interferon alfa-2a, or both in metastatic renal-cell carcinoma. Groupe Francais d’Immunotherapie. N. Engl. J. Med..

[119] Alsharedi M., Katz H. (2018). Check point inhibitors a new era in renal cell carcinoma treatment. Med. Oncol..

[120] Motzer R.J., Escudier B., McDermott D.F., George S., Hammers H.J., Srinivas S., Tykodi S.S., Sosman J.A., Procopio G., Plimack E.R. (2015). Nivolumab versus Everolimus in Advanced Renal-Cell Carcinoma. N. Engl. J. Med..

[121] McDermott D.F., Atkins M.B. (2013). PD-1 as a potential target in cancer therapy. Cancer Med..

[122] Siska P.J., Kim B., Ji X., Hoeksema M.D., Massion P.P., Beckermann K.E., Wu J., Chi J.T., Hong J., Rathmell J.C. (2016). Fluorescence-based measurement of cystine uptake through xCT shows requirement for ROS detoxification in activated lymphocytes. J. Immunol. Methods.

[123] Li Y., Zhu B. (2018). Editorial: Metabolism of Cancer Cells and Immune Cells in the Tumor Microenvironment. Front. Immunol..

[124] Nakaya M., Xiao Y., Zhou X., Chang J.H., Chang M., Cheng X., Blonska M., Lin X., Sun S.C. (2014). Inflammatory T cell responses rely on amino acid transporter ASCT2 facilitation of glutamine uptake and mTORC1 kinase activation. Immunity.

[125] Klysz D., Tai X., Robert P.A., Craveiro M., Cretenet G., Oburoglu L., Mongellaz C., Floess S., Fritz V., Matias M.I. (2015). Glutamine-dependent α-ketoglutarate production regulates the balance between T helper 1 cell and regulatory T cell generation. Sci. Signal..

[126] Crawford J., Cohen H.J. (1985). The essential role of L-glutamine in lymphocyte differentiation in vitro. J. Cell. Physiol..

[127] Fraternale A., Brundu S., Magnani M. (2017). Glutathione and glutathione derivatives in immunotherapy. Biol. Chem..

[128] Gamcsik M.P., Kasibhatla M.S., Teeter S.D., Colvin O.M. (2012). Glutathione levels in human tumors. Biomarkers.

